# Label-free metabolic imaging of *Pseudomonas aeruginosa* infection using two-photon fluorescence lifetime imaging microscopy

**DOI:** 10.3389/fcimb.2025.1642009

**Published:** 2025-09-22

**Authors:** Greg A. Snyder, Tialfi Bergamin de Castro, Alison J. Scott, Krishanu Ray

**Affiliations:** ^1^ Division of Vaccine Research, Institute of Human Virology, University of Maryland School of Medicine, Baltimore, MD, United States; ^2^ Department of Microbiology and Immunology, University of Maryland School of Medicine, Baltimore, MD, United States; ^3^ University of Maryland School of Medicine, Baltimore, MD, UnitedStates; ^4^ Department of Microbial Pathogenesis, University of Maryland School of Dentistry, Baltimore, MD, United States; ^5^ Department of Biochemistry and Molecular Biology, University of Maryland School of Medicine, Baltimore, MD, United States

**Keywords:** NAD(P)H, metabolism, bacteria, infected cells, infected tissue, oxidative phosphorylation, two-photon fluorescence lifetime imaging microscopy

## Abstract

In this study we have applied high-spatial and temporal label-free imaging of individual live multidrug-resistant bacteria and bacteria-infected cells and animal tissue using two-photon fluorescence lifetime imaging microscopy (2p-FLIM). 2p-FLIM can identify and quantify fluorescence intensity and lifetimes among bacteria, infected cells, and tissues. We have implemented 2p-FLIM in combination with phasor plot analysis for quantifying molecular differences of NAD(P)H intensities and lifetimes for fast, sensitive, high-resolution, non-destructive imaging of live bacteria and bacteria-infected cells and tissues. We have further developed a coordinated workflow using 2p-FLIM for high-resolution temporal-spatial mapping of bacteria infected cells and tissues that can be performed for near real-time quantitation of NAD(P)H intensities and lifetimes for identifying changes in metabolism. 2p-FLIM may have broad applicability for characterizing microbial infection at the molecular, subcellular, cellular and tissue levels. The ability to quantitate and directly monitor changes in NAD(P)H metabolism in near real-time in bacteria cells and tissues during an infection, offers a potential mechanism for understanding microbial pathogenesis and evaluating therapeutic treatments that can be applied to multiple model systems. Overall, the application of this label-free imaging approach has the potential to address biomedical research needs and technical problems that occur broadly across multiple biological systems and diseases.

## Introduction

Understanding the intricate mechanisms by which microbes modulate host immunity and metabolism is important for elucidating infection, survival, propagation, pathogenesis, and even the maintenance of immune homeostasis. To better understand and characterize microbial-host infections, we have developed quantitative non-destructive label-free imaging of individual bacteria and bacteria-infected cells and tissue. Label-free molecular imaging allows for near real-time quantitative assessment and characterization of microbial infection. 2p-FLIM is a label-free biological imaging technique increasingly used in biomedical and healthcare applications (Mongeon et al., 2016; Masia et al., 2018; Ranawat et al., 2019).

2p-FLIM has been primarily used in cancer biology and recently, in hospital surgical settings to image metabolically altered cells for determining the boundary and margins of metabolically altered tumors and tissues (Sun et al., 2010; Kolenc and Quinn, 2019). It is becoming more widely used for quantitative studies of cellular functions and biomedical applications, including tissue morphology and high-density protein arrays (Skala et al., 2007; Sun et al., 2010; Sun et al., 2012; Coda et al., 2014; Sarder et al., 2015). However, the application of 2p-FLIM for evaluating microbial infection and associated cellular perturbations has not been fully realized (Bhattacharjee et al., 2017; Sapermsap et al., 2020). To better characterize microbial infection, we have employed the label-free 2p-FLIM method for characterizing and quantitating changes in bacteria and bacteria-infected cells and tissues. The intrinsic fluorescence of 1,4-dihydro nicotinamide adenine dinucleotide (NADH) and its phosphorylated form NADPH – collectively referred to as NAD(P)H, which is detected by 2p-FLIM, provides a unique spectral wavelength of high transmittance for monitoring shifts in metabolism involving bacterial systems (Kolenc and Quinn, 2019; Perinbam et al., 2020). Individual bacteria and infected hosts have changes in NAD and associated metabolism (Coronas-Serna et al., 2020; de Sousa et al., 2021). 2p-FLIM provides high-resolution cellular and subcellular imaging of NAD(P)H total fluorescence and lifetimes in live cells. Conventional steady-state methods are frequently inadequate for the quantitative investigation of cellular function at the molecular level (Berezin and AChilefu, 2010). 2p-FLIM uses the intrinsic autofluorescence of NADH and NADPH present in microbes and host cells and monitors changes in their fluorescence intensity and lifetimes within biological conditions. The autofluorescence intensity and lifetimes of NADH and NADPH can be used as a measure of cellular metabolism (Schaefer et al., 2019; Cao et al., 2020). Cellular NAD+/NADH ratios are estimated at ~700:1 (Fjeld et al., 2003). Therefore, discrete alterations in cellular NAD+ are likely to be reflected as significant changes in NADH. Additionally, differences in fluorescence lifetimes (FLT) of either free and protein-bound forms of NADH or NADPH are independent of intensity (Schaefer et al., 2019). Label-free quantitation of endogenous NAD(P)H fluorescence and NAD(P)H-associated metabolites provides high-spatial and temporal imaging of bacteria and infected tissue at single organism, tissue, cellular, sub-cellular and molecular levels, which can provide a mechanistic view of the bacterial-host interactions. The relationship between microbial infection and cell metabolism can be used for understanding bacteria and treating infection (Valle-Casuso et al., 2019). Furthermore, the implementation of phasor analyses approach to the FLIM data can generate an excellent visual qualitative/quantitative demonstration of the metabolic process in the live cells and tissues. We have previously shown that 2p-FLIM can be used to characterize virally induced metabolic changes in HIV-1 infected cells and tissues (Snyder et al., 2023). To expand on these studies, we have further developed 2p-FLIM for evaluating metabolic differences of both individual bacteria and bacteria-infected cells and tissues showing that 2p-FLIM can used to evaluate live bacterial infection of cells and tissue including subcellular and molecular level using the same individual or serially sectioned infected sample.

We have selected *Pseudomonas aeruginosa (Pa)* because *Pa* strains in different environments exhibit a broad range of metabolic activity among *in vitro* and *in vivo* host-adapted strains, for which we have a previously developed infection model (Scott et al., 2019; Riquelme et al., 2020; Riquelme and Prince, 2021). Pa is an opportunistic Gram-negative multidrug-resistant (MDR) bacterium that affects individuals in healthcare settings, immunocompromised, with burn wounds and with cystic fibrosis (CF). Complications resulting from *Pa* infection can lead to serious lung infection (pneumonia), injury and death. The PAO1 strain represents a widely characterized *in vitro* laboratory reference strain of *Pseudomonas* (Stover et al., 2000). Clinical isolates CF63 and CF1188 represent *in vivo* host-adapted strains from the lung of individuals with cystic fibrosis (Burns et al., 2001; Brao et al., 2020; Horspool et al., 2023). Understanding the mechanisms by which *Pa* interacts with the host’s immune system and manipulates host metabolism is crucial for comprehending its ability to establish infection, survive within the host, multiply, and ultimately cause disease.

In this paper, we advance label-free quantitative 2p-FLIM and phasor analyses to investigate differences in NAD(P)H of *in vitro* and host-adapted *Pa* strains and infected cells and cryo-sectioned tissues harvested from a lung infection mouse model. The ability to quantify label-free metabolic changes in real-time using live pathogenic bacteria of different strains and infected host cells and tissues offers a dynamic and unique approach for evaluating bacterial virulence at the tissue, cellular, subcellular, and molecular levels. 2p-FLIM is particularly useful for characterizing individual metabolites (like NAD(P)H) and metabolic pathways (like glycolysis and oxidative phosphorylation). We observe an increase in the enzyme-bound NAD(P)H fraction in infected cells and tissues, indicating the upregulation of oxidative phosphorylation compared to uninfected controls. This approach allows for the visualization and quantification of metabolic states of bacteria and the host during infection.

## Materials and methods

### Bacteria

*Pa* laboratory reference strain PAO1, CF63, and CF1188 were provided by Robert Ernst. CF63 is an isolate from a person with cystic fibrosis (pwCF). *Pseudomonas aeruginosa* PAO1 and CF63 (University, 2017; HOLLOWAY, 1955; Stover et al., 2000; Klockgether et al., 2010) were streaked onto a Luria broth (LB) agar growth media plate and grown overnight at 37°C. A single colony from each plate was then inoculated into a 14 milliliters (ml) round bottom tube containing 4ml of LB and grown overnight (~12-15hrs) at 37°C with 225rpm shaking. An aliquot of this overnight growth was inoculated to a new tube containing 4 mL LB and adjusted until the optical density at 600 nanometers was 0.1-.15 (O.D.600nm). Bacteria were grown @ 37°C with shaking (225rpm) until the solution measured 0.5 at O.D. 600nm. An aliquot was taken adjusting for small differences in optical density to represent a similar number of bacteria for each PAO1 or CF63. Bacteria were centrifuged at 1000 rpm for 4 minutes at 4°C, washed two times with PBS, resuspended in 500μl PBS and then 10μl volume was aliquoted onto a lysine coated glass chambered slide.

### Bacteria and A549 coincubation

A549 cells (ATCC- CC-185) grown in phenol red-free Dulbecco’s modified Eagle’s medium (DMEM) F12K supplemented with 10% heat-inactivated fetal bovine serum (Gibco) and 1% l-glutamine (Gibco) without antibiotics at 1 x10^4/ml in a chambered glass slide (Lab-Tek) was incubated with bacteria at O.D._600_ 0.4 for ~1 hour at 37°C, 5% CO2.

### Animals

Adult, female C57BL/6J mice (Jackson Laboratories, Bar Harbor, ME) were housed in a pathogen free, particle-filtered negative pressure airflow caging system. Food and water were provided ad libitum. Mice were euthanized by carbon dioxide narcosis followed by thoracotomy during the inflation procedure. All animal procedures were performed under an approved protocol administered by the Institutional Animal Care and Use Committee at the University of Maryland, Baltimore.

### Mouse lung infection and inflation

C57BL/6 mice were infected intranasally (i.n.) with CF1188 10^7 CFU and lung tissues were prepared 48 hours post-infection according to our previously published method (Scott et al., 2019). Gelatin-inflated lung cryosections previously developed for MSI (Yang et al., 2020) were tested and found to be compatible with 2p-FLIM. Serial sections were thaw-mounted onto coverslips for 2p-FLIM measurements.

### 2p-FLIM

PBS washed bacteria were aliquoted to a custom chambered polylysine coated slide and sealed with tape. Sealed polylysine coated chambered slides were incubated for ~10min to allow bacteria to settle and attach and then imaged using 2p-FLIM. A customized confocal microscope (based on ISS Q2 laser scanning nanoscope) with single-molecule detection sensitivity was used for performing 2p-FLIM. A detailed description of this microscope setup is previously described (Snyder et al., 2023). Imaging was performed upon 2p excitation at 780 nm by 90fs pulsed laser and detection of NAD(P)H in the 435–485 nm spectral range. Time-resolved fluorescence data were fitted with bi-exponential model. All time-resolved fluorescence data were analyzed using ISS VistaVision software. The values of FLTs (τ_1_ and τ_2_) were obtained using the ISS vistavision software with the deconvolution of instrument response function and nonlinear least-squares fitting. Fluorescence intensity images are shown in green with kilo counts per sec (KCPS) scaled as vertical bars. Corresponding fluorescence lifetime images (nanosecond, nsec) are presented. Linear red-green-blue (RGB) colored scale bars indicating changes in fluorescence lifetimes (FLTs) between 0 to 6 nsec range are displayed for each FLIM image panel.

### Phasor analyses

The phasor is a visual demonstration of the raw FLIM data in a vector space, named as the phasor space. The phasor plot selects the raw data from each image pixel and converts it into a single point in the phasor space. This space uses two phasor vectors G and S (defined by Equation 1), to represent the location of each data point.


(1) $$g\left(\omega \right)=\frac{\int_{0}^{\infty }I\left(t\right)\cos\left(\omega t\right)dt}{\int_{0}^{\infty }I\left(t\right)dt};\;\;s\left(\omega \right)=\frac{\int_{0}^{\infty }I\left(t\right)\sin\left(\omega t\right)dt}{\int_{0}^{\infty }I\left(t\right)dt}$$

where g and s are horizontal and vertical axes of the phasor plots, and ω is the modulation frequency. Each pixel in the image is mapped to the phasor plots at a specific (G, S) coordinate, called the phasor point, which gives the fluorescence lifetime information of the pixel; the number of pixels mapped to the same phasor point is indicated by the brightness of the phasor point. Each phasor point on the phasor plots provide directly two pieces of information: its location given by the (G, S) coordinate represents the unique fluorescence lifetime species (single- or multi- exponential), and its brightness indicates how many pixels in the image are mapped to the phasor point for the same fluorescence lifetime species.

## Results

### Study design using 2p-FLIM for evaluating metabolic differences of individual bacteria and bacteria-infected cells and tissue

**Figure 1:**
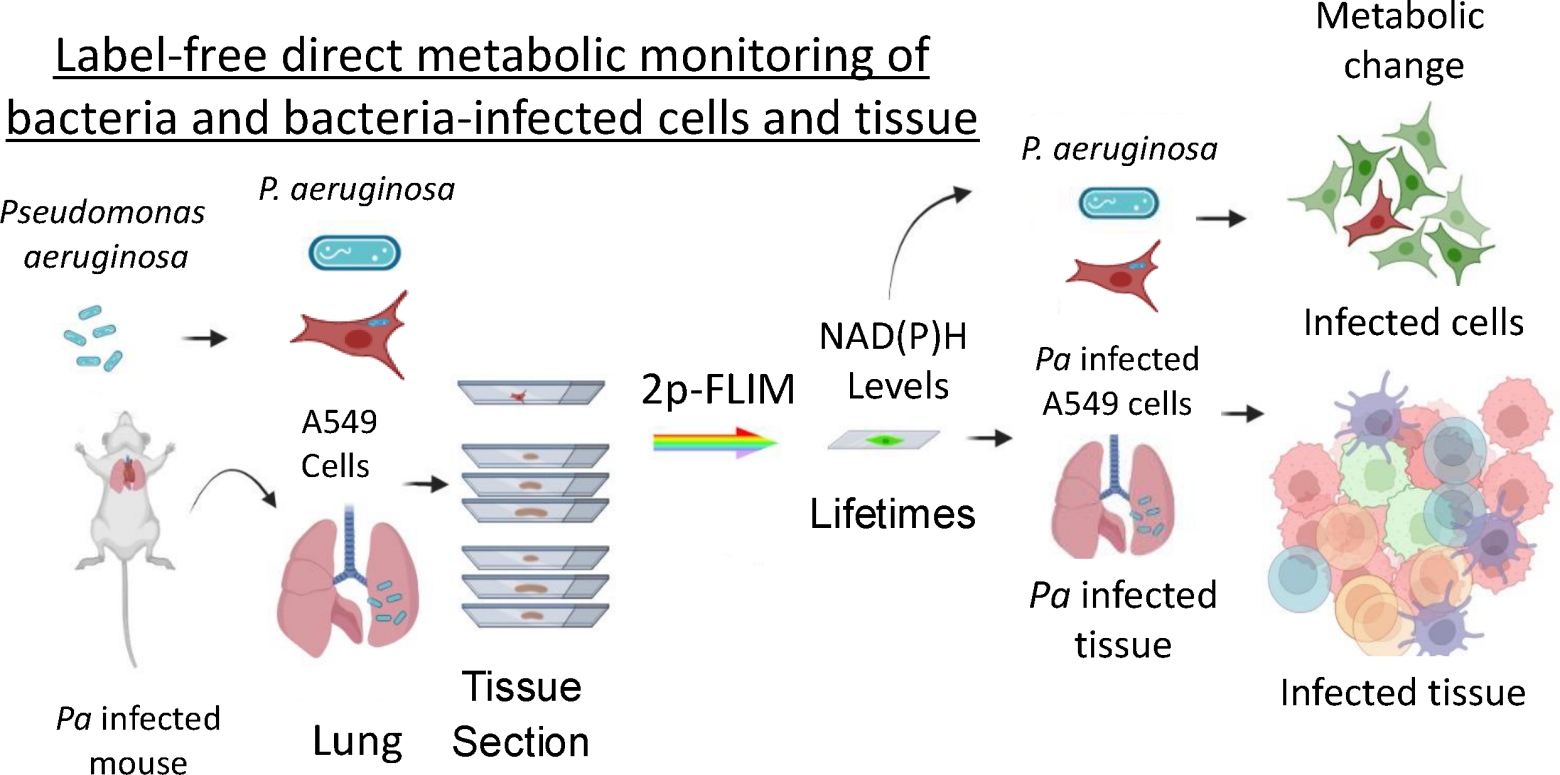
Summary of infection model and label-free methodology. Live cells and individual bacteria from different strains were used for a label-free imaging method – 2p-FLIM. The *Pa* bacteria mouse lung infection model was used for developing 2p-FLIM.

We have previously shown that 2p-FLIM can be used to characterize metabolic changes in HIV-1 infected cells and tissues (Snyder et al., 2023). To expand these studies, we have further developed 2p-FLIM for evaluating metabolic differences of individual bacteria and bacteria-infected cells and tissues that can be used to evaluate live bacteria, infected cells and tissue at the cellular, subcellular and molecular level on the same individual or serially cryo-sectioned tissue samples (**Figure 1**).

### 2p-FLIM identifies differences in NAD(P)H fluorescence intensities and lifetimes among bacteria

**Figure 2:**
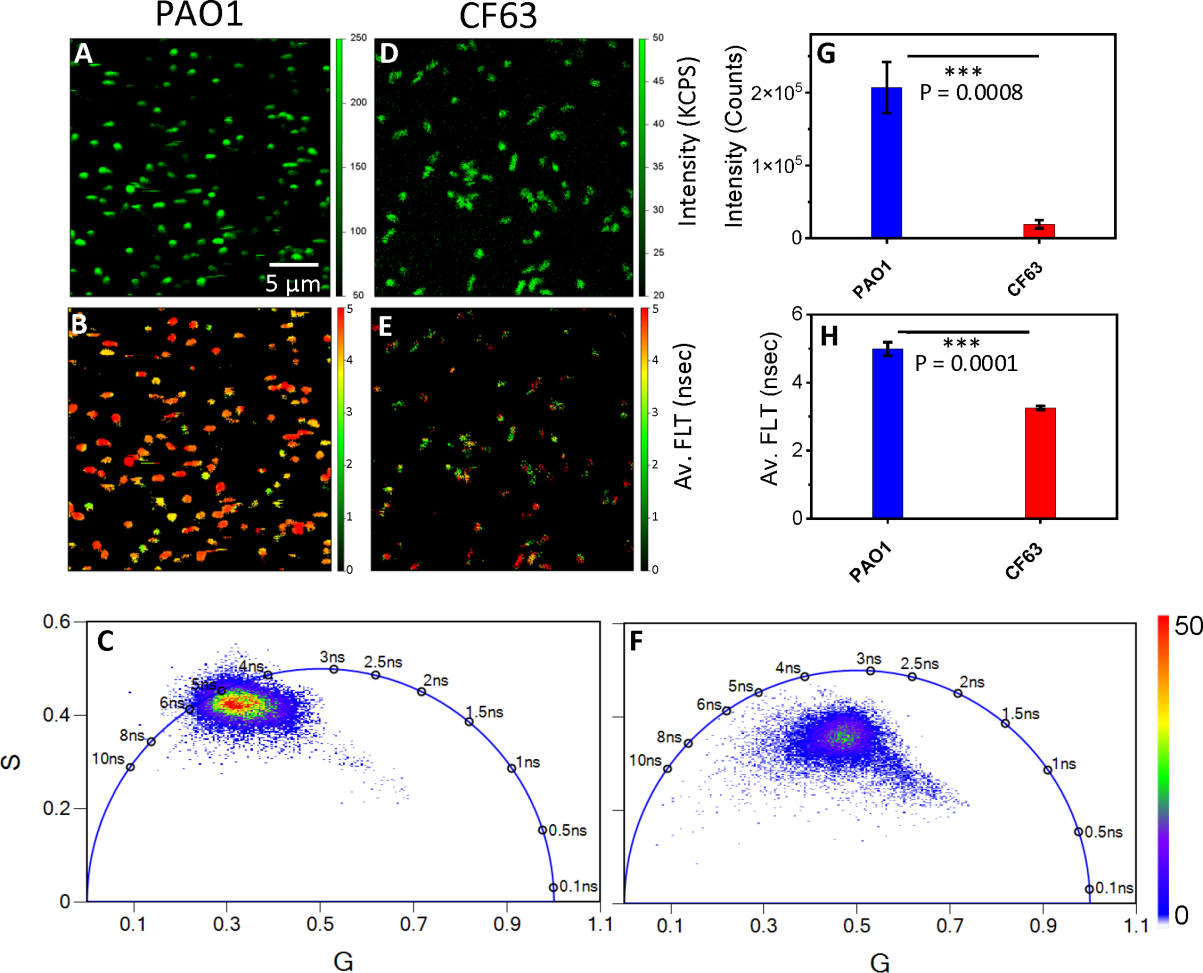
2p-FLIM identifies differences in NAD(P)H fluorescence intensity and lifetimes of Pa bacteria PAO1 and CF63. Label-free live 2p-FLIM of NAD(P)H of PAO1 (panels **A–C**) and CF63 (panels **D–F**). Panels **(A)** PAO1 and **(D)** CF63 show the fluorescence intensity of NAD(P)H. Panels **(B)** and **(E)** show the average FLT of NAD(P)H from PAO1 and CF63. Quantitative differences of NAD(P)H intensities and FLTs **(G, H)** for PAO1 and CF63 are shown as bar graphs. FLTs recorded by 2p-FLIM were depicted as phasor Plots in **(C, F)**. PAO1 phasor profile displays longer FLT compared to CF63.

Using 2p-FLIM, we have performed live label-free imaging of a widely used laboratory reference strain of *P. aeruginosa* (PAO1) in comparison with pwCF (University, 2017; HOLLOWAY, 1955; Stover et al., 2000; Klockgether et al., 2010). PAO1 is a widely used laboratory reference strain originally isolated from burn wound, while CF63 was isolated from an individual with severe cystic fibrosis (CF) and yields a high-burden of infection in the lungs of mice in a CF model (Brao et al., 2020). We observe differences in NAD(P)H fluorescence intensity among the two bacterial strains (PAO1 and CF63). PAO1 bacteria exhibited higher fluorescence intensities in comparison with CF63 (**Figures 2A, D**). This is exhibited by a nearly tenfold difference in kilo counts per second (KCPS) in the vertical intensity scale bar between PAO1 and CF63. When quantitated, a 5-fold increase in NAD(P)H fluorescence intensity for PAO1 bacteria is observed compared with CF63 (**Figure 2G**). Interestingly, more PAO1 were attached to the poly-lysine coated coverslips compared to CF63 (**Figures 2A, D**). FLT is independent of probe (NAD(P)H - for this study) concentration, so any changes observed in the protein bound form of NAD(P)H (τ_2_) represent the inherent changes in molecular properties. In cells, NAD(P)H exists in both free and protein bound forms, which have almost identical spectra but very different lifetimes. Correspondingly, all the intensity decays and FLIM images were fit with a bi-exponential model. Using a bi-exponential fit, the intensity decay of NAD(P)H from PAO1 yielded a τ_1_ value of 1.13 nsec (f_1_ = 0.09) and τ_2_ value of 5.12 nsec (f_2_ = 0.91) with a χ^2^ value of 1.38. Similarly, the bi-exponential fit to the intensity decay of NAD(P)H from CF63 exhibited a τ_1_ value of 0. 6 nsec (f_1_ = 0.18) and τ_2_ value of 3.74 nsec (f_2_ = 0.82) with a χ^2^ value of 1.18. PAO1 exhibits longer average FLTs when compared with CF63 (**Figures 2B, E**), indicating differences mainly in the bound forms of NAD(P)H among each bacteria type. When quantitated, a significant increase in average NAD(P)H fluorescence lifetime for PAO1 bacteria is observed compared with CF63 (**Figure 2H**). Differences in FLTs can be further observed when plotting the distribution pattern of individual FLTs as represented by phasor plot profiles (Leben et al., 2019). The phasor plots of 2p-FLIM represent distinctive FLT profiles for each bacteria set (**Figures 2C, F**). The single-exponential FLT species fall on the semicircle of the phasor plots and the FLT value is directly given by the phasor plots. However, in case of NAD(P)H fluorescence in bacteria, like intensity decays, the phasor plots clearly show the presence of two species – NAD(P)H in free form and NAD(P)H in bound to enzymes. Furthermore, a compact and left shift is observed for PAO1 FLT population compared with a more diffuse central distribution of FLTs for CF63 (**Figure 2F**). This observation in phasor plot profiles reflects the differences in metabolism between PAO1 and CF63. Additionally, the two-tailed t-test revealed a significant difference in both intensity and FLT of NAD(P)H between PAO1 and CF63 strains.

### 2p-FLIM identifies differences in NAD(P)H fluorescence intensities and lifetimes in bacteria infected A549 cells

**Figure 3:**
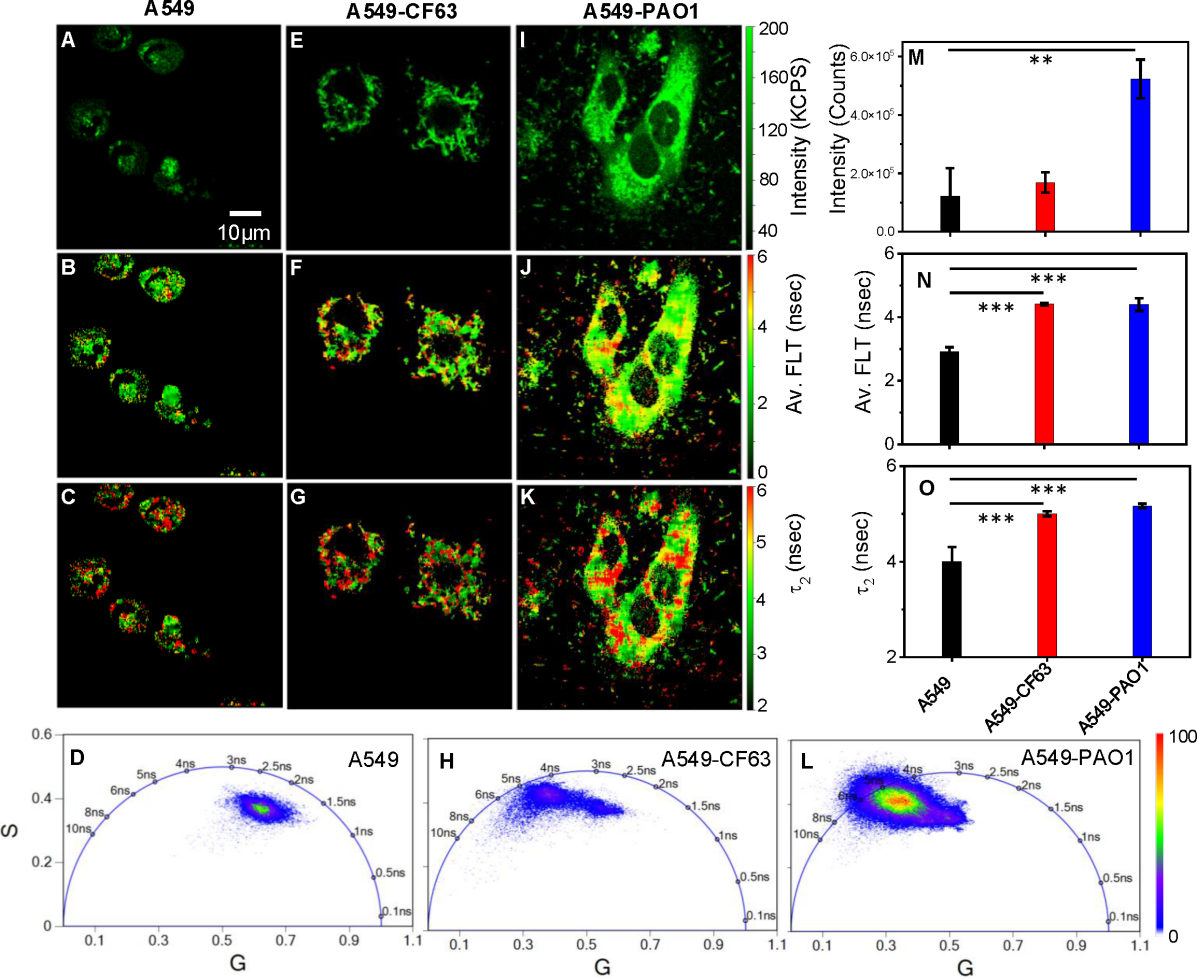
PA-specific differences in NAD(P)H fluorescence intensity and lifetimes. Label-free live 2p-FLIM of NAD(P)H with A549 cells upon infection with *Pa* strains. Differences in NAD(P)H fluorescence intensity **(A, E, I)**, average FLT of NAD(P)H **(B, F, J)**, and bound NAD(P)H FLT **(C, G, K)** for A549 cells infected with *Pa* compared to A549 cell control. Phasor plots of NADH fluorescence from A549 cells **(D)** and after infection with *Pa* CF63 **(H)** and PAO1 **(L)**. The phasor histogram of the cell transfected with bacteria are shifted to the direction of bound form of NAD(P)H. Image intensity, average FLT, and bound form of NAD(P)H FLTs are quantified in bar graphs **(M–O)**. All experiments were repeated with the mean values shown. Error bars indicate standard deviations. Two-tailed test was performed for statistical analysis in **(M–O)**, P values; **<0.01 and ***< 0.001.

Label-free live 2p-FLIM imaging of NAD(P)H in A549 cells upon infection with CF63 or PAO1 bacteria is shown (**Figure 3**). A significant change in NAD(P)H intensity, average FLT, and FLT in the bound form of NAD(P)H in A549 cells upon incubation with bacteria compared to A549 control is observed and quantified in bar graphs (**Figures 3M–O**). As described earlier, NAD(P)H in cells shows short FLT (<1 ns) and long FLT (>2.5 ns), corresponding to free and protein-bound forms of NAD(P)H in cells. Thus, cellular NAD(P)H lifetime data are typically analyzed by double-exponential decay fitting to resolve free (fast component) versus bound (slow component) forms. Using a bi-exponential fit, the intensity decay of NAD(P)H from uninfected A549 cells yielded a τ_1_ value of 0.89 nsec (f_1_ = 0.34) and τ_2_ value of 3.6 nsec (f_2_ = 0.66) with a χ^2^ value of 1.07. In contrast, the bi-exponential fit with the intensity decay of NAD(P)H from CF63 infected A549 cells exhibited a τ_1_ value of 0.96 nsec (f_1_ = 0.3) and τ_2_ value of 4.04 nsec (f_2_ = 0.7) with a χ^2^ value of 1.05. Similarly, the fit to the intensity decay of NAD(P)H from PAO1-infected A549 cells exhibited a τ_1_ value of 1.02 nsec (f_1_ = 0.37) and τ_2_ value of 4.2 nsec (f_2_ = 0.63) with a χ^2^ value of 1.13. The χ^2^ values for all the fits to the different intensity decay data sets were in the range of 0.96 to 1.13, indicating an overall good fit to the experimental data. With either CF63 or PAO1 infection, we observed enhanced fluorescence intensity of NAD(P)H and long FLT associated with the bound form of NAD(P)H compared to uninfected A549 cells as displayed in (**Figure 3**: NAD(P)H fluorescence intensity (A, E, I), average FLT of NAD(P)H (B, F, J), bound NAD(P)H FLT (C, G, K) for A549 cells infected with CF63 (E, F, G) or PAO1(I, J, K) compared to A549 (A, B, C) cell control). Corresponding Phasor plots of NAD(P)H fluorescence from A549 cells and after infection with CF63 and PAO1 are shown (**Figures 3D, H, L**, respectively). The two-tailed t-test revealed a significant difference in both intensity and FLT of NAD(P)H between PAO1 infected and uninfected A549 cells (**Figures 3M-O**). **Figures 3N, O** were constructed using data from the entire system, encompassing both the bacteria and the A549 cells. Considering the phasor coordinates of the lifetime of the free NAD(P)H and the bound form of NAD(P)H, the phasor plots for A549 cells with or without bacteria fall under the semicircle, suggesting the presence of both forms of NAD(P)H. It is apparent from the phasor plot that upon infection, the population shifts significantly to the left (longer FLT scale), indicating a higher oxidative phosphorylation state (Perinbam et al., 2020). Although, the bar graphs do not show a statistically significant difference in average lifetimes, the FLT images (**Figure 3F** vs J; G vs K) display different spatio-temporal distributions and phasor plots (**Figures 3H, L**) demonstrate significant differences in the distribution of NAD(P)H between CF63 and PAO1 infected A549 cells. Additionally, we observed an increase in the NAD(P)H bound lifetime for PAO1 infected cells (4.2 ns) compared to CF63 infected cells (4 ns).

### Differences in NAD(P)H fluorescence intensity and FLTs among Pa CF1188 infected mouse lung tissue and control tissue sections are identified using 2p-FLIM

**Figure 4:**
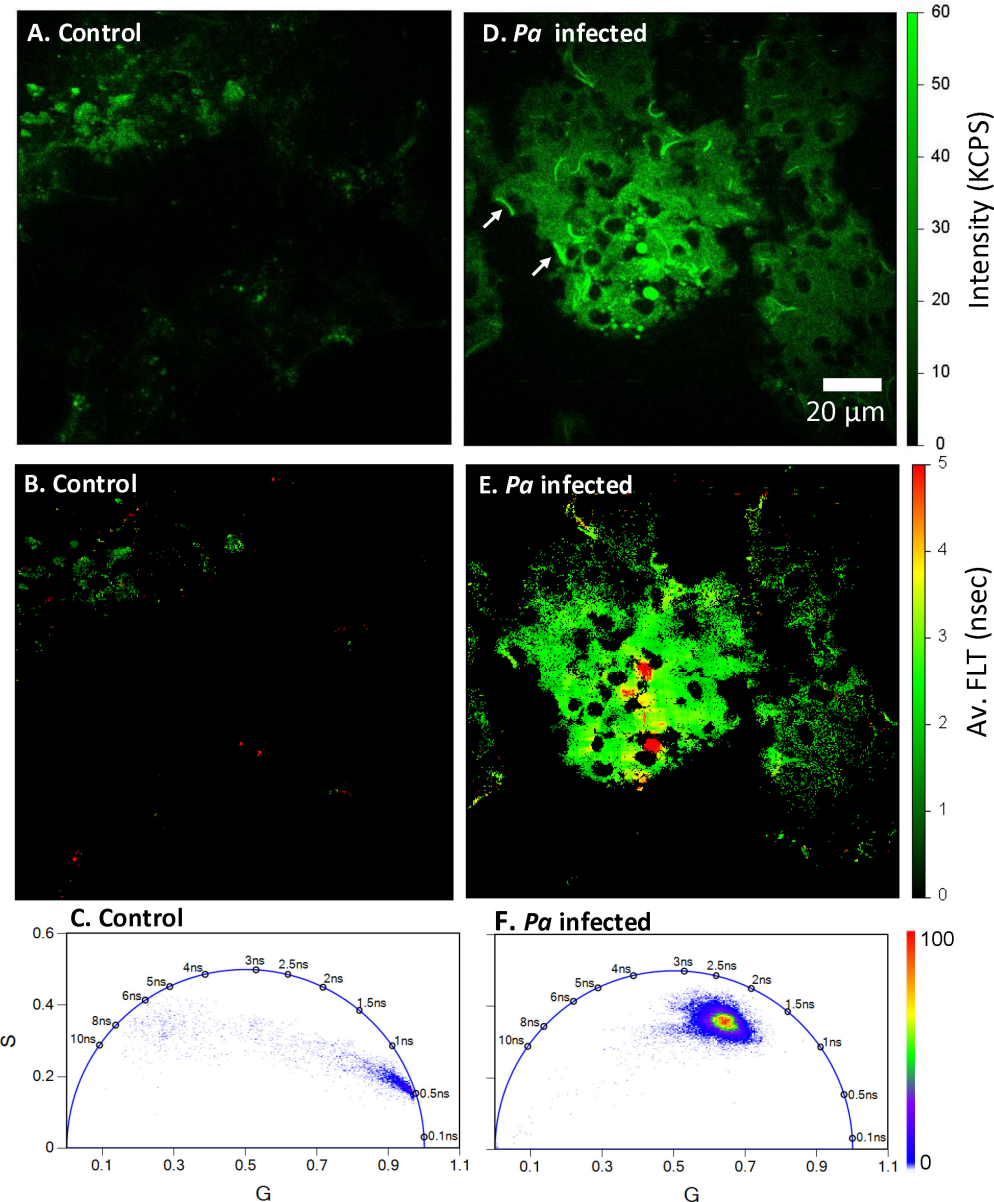
Bacteria-specific differences in NAD(P)H fluorescence intensity and lifetimes. Label-free 2p-FLIM of NAD(P)H in uninfected (panels **A–C**) and *Pa* CF1188 infected (panels **D–F**) cryo-sectioned (13 µm thick) lung tissue from mouse. Panel **(D)** shows the NAD(P)H fluorescence signals from CF1188 infected tissue. Fluorescence from *Pa* are identified as white arrows. Panel **(E)** shows the average FLT of NAD(P)H in the infected lung tissue section. FLIM FLTs using the Phasor Plots in **(C, F)** displays longer lifetime for Pa infected mouse lung tissue compared to uninfected control tissue sections.

To better understand how bacterial infection affects tissue, we have further characterized Pa-infected mouse lung tissues using 2p-FLIM. Cryo-sectioned mouse lung tissue exposed to mock-infected control or *Pa* strain CF1188 was imaged using 2p-FLIM 48 hours post-intranasal infection. The CF1188 strain is a pediatric clinical isolate from a person with Cystic Fibrosis (pwCF) that has a mucoid phenotype (Burns et al., 2001; Brao et al., 2020). Our data show that gelatin-inflated lung cryosections are compatible with 2p-FLIM. NAD(P)H fluorescence and average FLTs of cryo-sectioned mouse lung tissue exposed to Pa were recorded using 2p-FLIM. An overall increase in NAD(P)H fluorescence intensity and FLT of Pa-infected lung tissue architecture is observed compared with mock-infected controls. The fluorescence intensity is consistent with bacteria shape and size, and bacteria-infected foci are discernable within infected lung tissue (**Figures 4A, D**; white arrows in panel 4D indicate bacteria). Additionally, differences in FLTs of Pa CF1188 infected lung tissue compared with mock-infected controls correlate with suspected infection foci (**Figures 4B, E**). **Figures 4C, F** show the phasor lifetimes of the control and *Pa* CF1188 infected mouse lung tissue sections, respectively. Our present study indicates an increase in FLT of NAD(P)H that is correlated with an increase in bound to free ratio of NAD(P)H in bacteria infected tissue sections. These changes indicate shifts towards oxidative phosphorylation (Perinbam et al., 2020).

## Discussion

**Figure 5:**
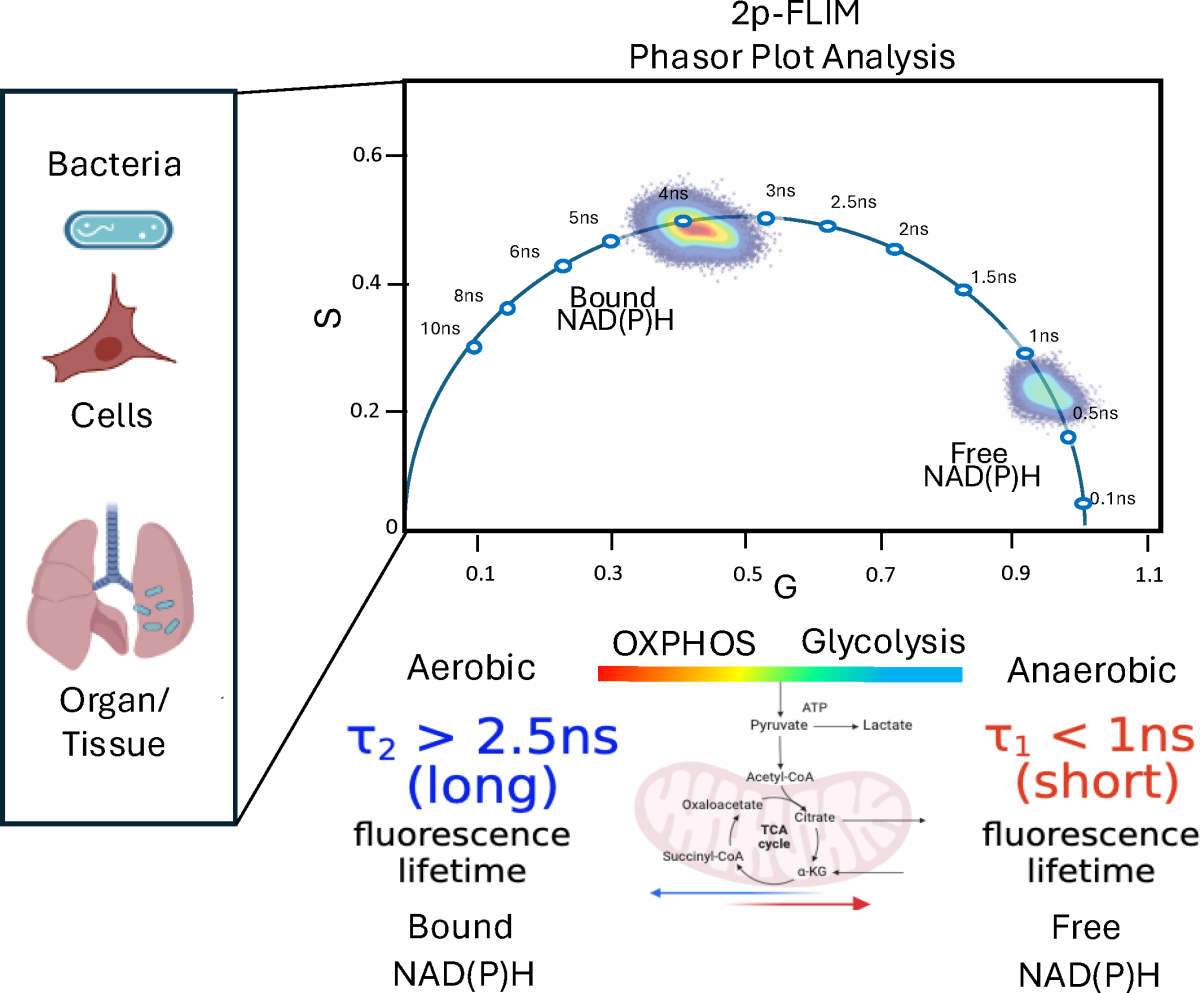
A depiction of cellular metabolism of bacteria and bacteria-infected cells and tissues by spatiotemporal 2p-FLIM. Graphic made using BioRender and Powerpoint.

2p-FLIM allows for highly sensitive and quantitative spatial and temporal monitoring of individual bacteria, associated metabolites and infected tissue. 2p-FLIM may be uniquely able to detect cellular dynamics of NAD(P)H levels and fluorescence lifetimes resulting from a bacterial pathogen infection. Recently there has been a growing interest in studying changes in metabolism for understanding how metabolic pathways work. NAD(P)H differences reflective of glycolysis or oxidative metabolism and those resulting from NAD(P)H metabolic disruptions, syndromes, or diseases can be observed (Naifeh et al., 2020; Smith et al., 2020; Giner et al., 2021). 2p-FLIM has the unique ability to resolve discrete molecular, subcellular as well as whole tissue NAD+ dynamics associated with glycolysis and oxidative phosphorylation metabolism resulting from microbial infection and NAD-associated metabolic dynamics (Naifeh et al., 2020; Smith et al., 2020; Giner et al., 2021). By measuring NAD(P)H fluorescence and lifetimes at the individual bacterium and single-cell level, we can provide visual evidence, localization, and quantitative information related to bacterial infection establishment, pathogenesis and potential interventions in a near real-time metabolic workflow using 2p-FLIM on different types of samples or tissue sections. 2p-FLIM fluorescence lifetimes and phasor plot analysis depicting metabolism of live bacteria, bacteria-infected cells and tissues is shown in **Figure 5**.

Both fluorescence intensity and lifetime-based NAD(P)H analyses show differences in individual bacteria, bacterial populations and bacteria-infected tissues. Changes in NAD(P)H fluorescence (intensity and FLT) provide information about the changes in NAD(P)H that have been correlated with metabolism (Ma et al., 2016). Fluorescent lifetime is independent of probe concentration, excitation intensity, and light scattering and can be uniquely sensitive to their molecular environment (NAD(P)H free or bound). Therefore, NAD(P)H fluorescence quantified within tissues can also reflect the extent to which NAD(P)H is either free or bound to a target. Moreover, fluorescent lifetime can be differentiated from background nonspecific autofluorescence, which often diminishes or complicates analysis. 2p-FLIM represents a dynamic label-free approach that is uniquely sensitive in assessing and quantitating dynamics of individual bacteria, bacterial populations and infected tissue at the diffraction-limited spatial resolution of ~300 nm.

We observe significant differences in NAD(P)H fluorescence intensities and lifetimes of individual bacteria, bacterial populations and bacteria-infected lung tissue as measured by 2p-FLIM. The bound form of NAD(P)H has a 5-times higher quantum yield than the free form of NAD(P)H (Fjeld et al., 2003; Ma et al., 2016; Schaefer et al., 2019). Furthermore, NAD(P)H lifetime varies significantly depending on if it is in a bound or free form. Changes in NAD(P)H lifetimes can be precisely displayed in a phasor plot to identify discrete metabolic changes individually and in populations (Leben et al., 2019). Specifically, the long fluorescence lifetimes of NAD(P)H observed in bacteria and infected tissue sections in the current study are due to the bound form of NAD(P)H. Based on this study and others, we imply that long FLT observed for NAD(P)H in infected cells and tissues is due to the bound form of NAD(P)H (Leben et al., 2019) (Sameni et al., 2016; Schaefer et al., 2019; Perinbam et al., 2020). Therefore, cellular NAD(P)H intensities and lifetimes associated with oxidative phosphorylation and infection can be quantitated, monitored, and compared with cells exhibiting glycolysis (free/unbound NAD(P)H shorter FLTs).

This study using a bacterial infection model, explores how NAD(P)H and fluorescence lifetime imaging could be used to identify unique signatures in cellular and tissue metabolism. Longer FLTs are linked to oxidative phosphorylation, while shorter FLTs are associated with glycolysis. 2p-FLIM has the potential to study how infections affect metabolism. The ability to actively monitor and quantitatively track bacteria-induced metabolic differences throughout infection using 2p-FLIM could provide new approaches for characterizing bacteria, bacterial infection and potential therapeutics. In mice, CF1188 has been used to model lung infection dynamics in both wildtype mice and in transgenic CF-like phenocopy mice (Scnn1b-Tg). The CF1188 isolate yields high bacterial burdens following intranasal infection, forms microcolony foci in the lung, and localizes in consolidated areas of the distal lung along with exhausted neutrophils (Brao et al., 2020). 2p-FLIM can monitor and characterize changes in both bacteria and host throughout an infection cycle or over treatment over the course of an infection (establishment, acute, chronic, biofilm formation, escape or resolution). Using phasor-FLIM approach, pixel maps of metabolic alteration in live bacteria, bacteria infected A549 cells and tissues were demonstrated. A significant shift towards the bound form of NAD(P)H, indicates an increase in oxidative phosphorylation for bacteria-infected cells and tissues. The phasor plots approach implemented to live cells and infected tissue section provides a convenient, easily interpretable interface to analyze the raw FLIM data both qualitatively and quantitatively. Further development of 2p-FLIM for label-free quantitative monitoring of infection systems could help to address technical and biomedical research across multiple biological systems or diseases.

In summary, the purpose of the current study was to investigate metabolic states of both individual bacteria, and bacteria infected cells and tissues using a microbial-host (pathogen-host) system. FLIM images provide a spatio-temporal distribution of a range of fluorescent lifetimes that correspond to different metabolic states. Host adapted pwCF strains exhibit differences in metabolism in comparison to reference PAO1 (Riquelme et al., 2020; Riquelme and Prince, 2021). Metabolic differences including itaconate usage could be expected to have differences in metabolism involving NAD, oxidative and glycolytic pathways. Combining 2p-FLIM with other methodologies can provide an integrative label-free quantitative multimodal microbial and molecular imaging of individual *Pa* bacteria and infected tissue providing a framework for proof of concept and expansion of these methodologies for microbial systems. A highly integrative, cross-platform, label-free live multi-imaging approach can provide a more complete molecular characterization of bacteria and its effect on infection establishment, propagation and pathogenesis within the context of authenticated pathogenic bacteria and infected host organisms. Integrated label-free molecular imaging studies using live pathogenic bacteria have the potential for assessing metabolic effects in a broad platform of live pathogenic, nonpathogenic, and commensal bacteria involved in pathogenesis, homeostasis, inflammation, and metabolic regulation. Since FLT and phasor plots are very sensitive and fluorescence decay times are independent of excitation intensity, fluorescence detection efficiency and local concentration of fluorophores, future studies will include a heat map of inhibitors exhibiting metabolic perturbations.

## Data Availability

The original contributions presented in the study are included in the article/supplementary material. Further inquiries can be directed to the corresponding author.
